# Supplementary figures and images for: Evaluation of two red cell inclusion staining methods for assessing spleen function among sickle cell disease patients in North-East Nigeria
Source: PLOS Glob Public Health. 2023 May 18;3(5):e0001552. doi: 10.1371/journal.pgph.0001552 (PMC10194925; doi:10.1371/journal.pgph.0001552)

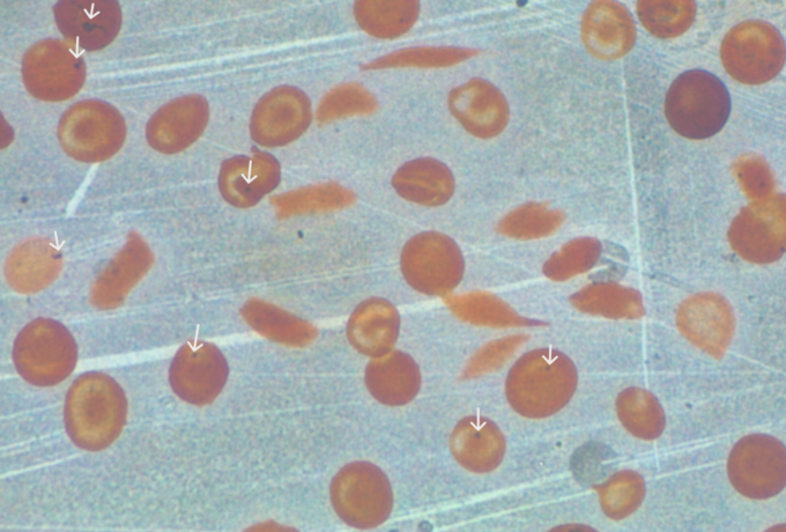

Supplement: S1 Fig — Silver-stained blood smear (x100/0.80) from a 22-year-old female HbSS patient showing deeply stained red cells (white arrows) making visualization of some of the argyrophilic inclusions difficult. (TIF) [file pgph.0001552.s001.tif]

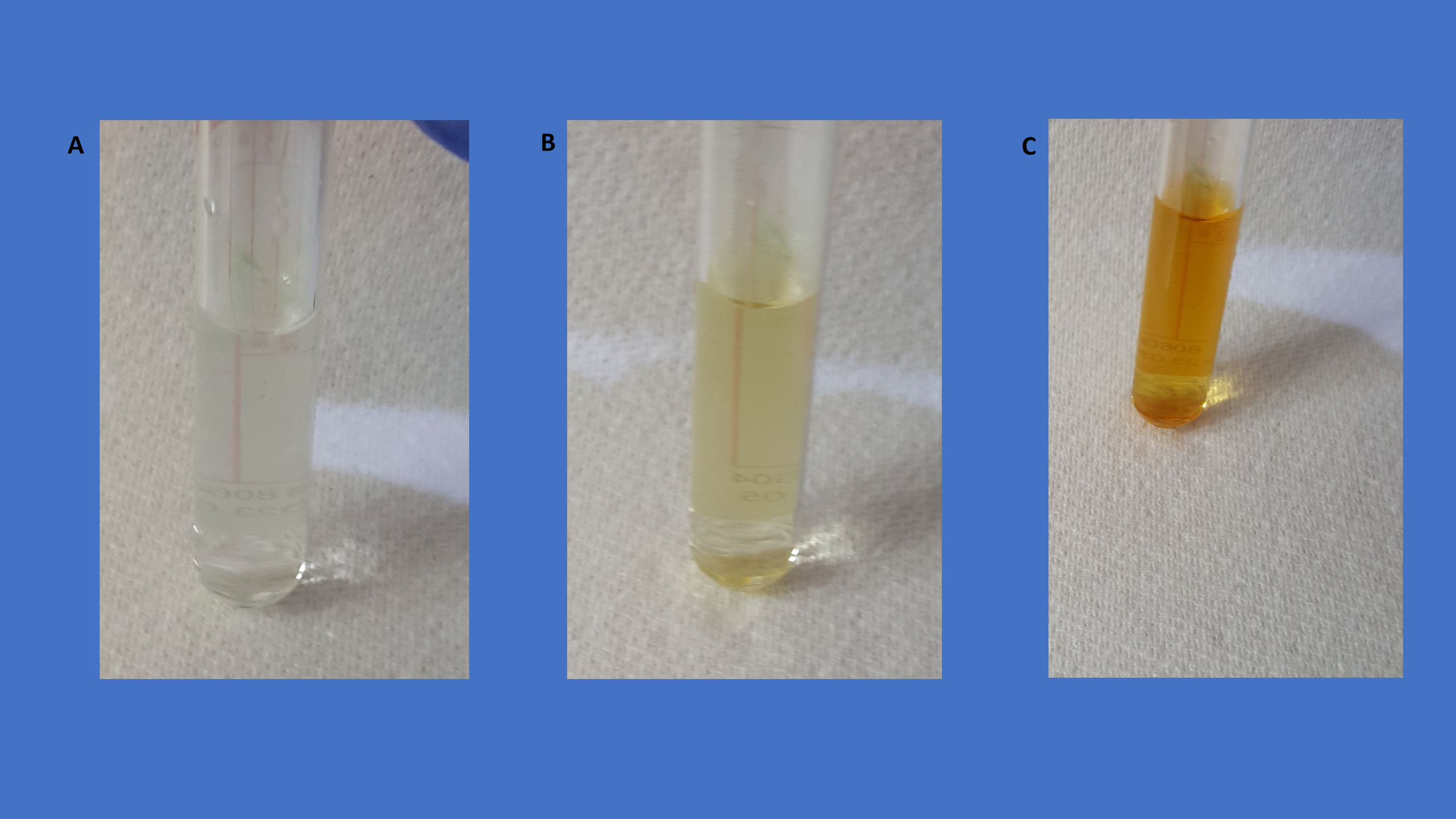

Supplement: S2 Fig — A) Silver stain at 1 minute. The preparation appears clear and colourless. B) The preparation has begun to take a yellow colouration. C) The preparation has turned golden yellow and continues deteriorating on further standing. (TIF) [file pgph.0001552.s002.tif]
