# Supplementary material for: Evaluation of two red cell inclusion staining methods for assessing spleen function among sickle cell disease patients in North-East Nigeria
Source: PLOS Glob Public Health. 2023 May 18;3(5):e0001552. doi: 10.1371/journal.pgph.0001552 (PMC10194925; doi:10.1371/journal.pgph.0001552)
Supplement: S1 Text — (DOCX) [file pgph.0001552.s003.docx]

# **Appendix: Standard operating procedure (SOP) - Silver staining of red cells**

**1.0 Purpose**

To describe the process of staining peripheral blood film using a silver stain for the identification of argyrophilic inclusions (AI) containing red cells.

**2.0 Principle**

This is based on the detection of erythrocytes containing argyrophilic inclusion demonstrated by silver stain. The silver stain was originally used to demonstrate the nucleolar organizer regions (NORs) of chromosomes, to evaluate their function, and to identify chromosomes in cytogenetic preparations (1-3). The technique was first applied by Tham and colleagues to red blood cells (4). The silver stain demonstrates all intracellular argyrophilic particles. The number of red cells containing argyrophilic inclusions (i.e AI) was directly related to the splenic reticuloendothelial function. The argyrophilic inclusions were shown to be Howell–Jolly bodies, Pappenheimer bodies and other inclusions visible in patients with a decreased or absent splenic function.

**3.0 Materials / reagents**

- Gelatin salt
- Silver nitrate salt
- Formic acid
- Potassium iodide
- Filter paper
- Formalin
- Ethanol
- Distilled water
- Beaker
- Eosin
- Glass slides
- Pipette
- Amber-glass bottles
- Microscope
- Incubator
- Paper towel
- Cover slips

**3.1 Staining reagents**

(i) Solution A (2%) - colloidal developer solution:

- Dissolve 2 g of Gelatin in 100 ml of distilled water and 1 ml of formic acid (1% v/v) at room temperature; requires at least 30 minutes at room temperature to dissolve
- Or continuous shaking at room temperature for 10 minutes
- Filter through Whatman filter paper.
- Store in room temperature for up to 6 months.

(ii) Solution B (50%), an aqueous silver nitrate solution:

- Dissolve 50g silver nitrate in distilled water in a proportion of 1:2 (w/v).
- Silver nitrate solution should be prepared just before use
- Or can be prepared beforehand and stored in refrigerator and protected from light. Store in capped amber-glass bottles or by other light-protective means.
- NB: Small-capped bottles should be used in which different quantities of silver nitrate like 1g, 2g, 3g or 4 g can be kept in small-capped bottle and stored away from light before use.
- Solution can be kept in fridge for up to 3 months

(iii) Working solution:

- Prepare fresh in acid-clean glassware just before use
- Take one part of solution A and mix with two parts solution B.
- Filter through Whatman filter paper into plastic bottle
- Only exact amount required is prepared (usually not more than 3mls for each batch of slides) because it degrades immediately.

**4.0 Method**

**4.1 Collection of blood sample:**

Aliquot of blood from an EDTA sample can be used for making the blood smear.

- 1. **Preparation of blood smears**
- Place a drop of blood near one end of the glass slide.
- Using a second slide to spread, draw the spreader backwards into the drop of blood at 45 degree or more depending on the haematocrit,
- Push forward in one quick movement. This should produce a film that is tongue shaped with a base, body, and a tip.
- Aim to make a smear not more than 3 cm in total length
- Label the slides with the subject ID and date one side of the slide.
- Air dry the slides.
- Smears can be fixed immediately or after a period of storage prior to silver staining.
- Air dried slides are fixed in 3:1 ratio of 95% ethanol to formalin mixture for 3 min (solution made by diluting 150 mL of formalin (37% formaldehyde) to 450 mL with 95% ethanol)
- The smears are washed with distilled water
- For immediate staining shake off water from slides as much as possible- blot to dry with towel
- For future staining, air dry the slides
- Store the slides in an airtight container.

**4.3 Staining of blood smears**

- Set up the incubator at least 10-15 minute to attain the desired temperature before staining.
- Just before staining, prepare the silver stain working solution (not more than 3 ml) as described above.
- Working quickly, cover the smear with two to three drops of freshly prepared working solution
- Gently drop a 40 X 24 mm coverslip on the slide to ensure even spread of the staining solution.
- Incubate in dark at 38^°^C for 20 min.
- Remove slides from incubator and working quickly remove cover slips and wash slides in several changes of tap water (distilled or deionized water can also be used)
- Counterstain with 1% eosin for 60 to 120 seconds.
- Wash in water.
- Dehydrate in increasing concentration of ethanol (i.e 70%, 90% and 100%)
- Dry slides in an incubator for about 10 minutes.
- Clear in xylene
- Mount

**4.4 Estimation of argyrophilic inclusion positive erythrocytes (AE)**

- Smears were examined using the x40/0.65 and oil immersion (x 100/0.80) HI PLAN objectives.
- Using a multiparameter counter tally, count 500 red cells. Note the number of red cells containing one or more distinct black inclusions and normal red cells per field.
- Express the number of AIs as a percentage of the total red cells.
- Only red cells with one or more distinct black granules are counted as positive. Red cells with a diffuse or a fine reticular or punctate pattern of brown staining are not regarded as positive for argyrophilic inclusions (may be due to the presence of RNA as indicated by polychromasia and punctate basophilia or retics count)

**4.5 Limitations:**

Slightly higher counts in patients with haemolytic anaemias may be due to the hematologic disturbance and not indicative of abnormal splenic function. Kent et al. found an increase in the percentage of red cells with autophagic vacuoles in patients with hematologic disturbance and reticulocytosis but intact spleens (5). A high proportion of reticulocytes contained vacuoles.

**Quality control**

1. **Pre test runs to check effect of several parameters so as to identify optimal staining parameters**
2. Reduction with potassium: reduction with potassium iodide is supposed to reduce background staining; a test run was conducted to check the effect of reduction with potassium iodide on the quality of slides. One batch of slides were pre-treated with potassium iodide and another batch from the same sets of participants were not pre-treated. Pre-treatment with KI did not produce any difference in the two group of slides, therefore this step of was removed from the final study protocol
3. Effects of temperature and staining duration: different batches of slides were stained either at room temperature or inside an incubator set at 38^°^ C or 40^°^C. Different time intervals was also applied to obtain the optimum staining time. Staining the slides at 338^°^C for 20 minutes gave the optimal quality of staining pattern. Staining at 338^°^C for 30 minutes (as described in the original protocol) or staining at higher temperature for 20 minutes resulted in over stained slides and formation of precipitates. Staining at room temperature required longer staining duration (almost an hour) and usually results in faintly stained slides which does not pick up the counterstain (eosin) adequately.
4. Different brand of silver nitrate salt: Two brands of silver nitrate salts were used. Both brands gave similar quality of staining reaction.
5. Eosin – The original protocol used an alcohol-based eosin. We compared this eosin and the traditional water-based eosin (eosin dissolved using distilled water); the latter gave a better quality of staining, the silver-stained inclusions were more distinct

## **Staining procedure**

1. For optimal slide quality, thin smears of not more than 3 cm were always prepared. Prior to staining, the slides were placed on a card-board slide holder/rack.
2. A maximum of 8 or 9 slides are placed on a single slide holder/rack, and the slides separated from each other by some inches to prevent overflow of the staining reagent from one slide onto the other.
3. Generally, not more than 2 or 3 drops of silver stain was placed on the centre of each smear. This prevented any overflow of excess stain, and helped to keep the slide racks clean, dry, and safe during the process of staining.
4. Only flat surface slide holders were used. This ensured the slides did not tilt and spill their content during the process of staining
5. The incubators were always set up to the optimal temperature of 38^°^C before the working solution is prepared.
6. Not more than 3mls of working solution is prepared per batch of slides, as the stain begins to deteriorate after that time and can no longer be used

## **Precaution**

1. Silver nitrate can cause severe skin burns and eye damage, general safety procedure including the use of personal protective equipment such as gloves and a laboratory coat was observed throughout the project.
2. The working area was always protected with paper towels.
3. The cover-slips removed from the stained slides staining were disposed of in a special disposal bag and separated from other lab waste.
4. The silver nitrate was always kept the in original container; during weighing out quantity required for a batch of working solution, care was taken not to create any aerosol

## **References**

1. Bukhari MH, Niazi S, Khan SA, Hashmi I, Perveen S, Qureshi SS, et al. Modified method of AgNOR staining for tissue and interpretation in histopathology. International journal of experimental pathology. 2007;88(1):47-53.

2. Lindner LE. Improvements in the silver-staining technique for nucleolar organizer regions (AgNOR). Journal of Histochemistry & Cytochemistry. 1993;41(3):439-45.

3. Ogunsola J, Antia R. Adaptation of the agyrophil technique for nucleolar organizer regions to canine peripheral blood smears. Open veterinary journal. 2018;8(2):182-5.

4. Tham KT, Teague MW, Howard CA, Chen SY. A simple splenic reticuloendothelial function test: counting erythrocytes with argyrophilic inclusions. American Journal Of Clinical Pathology. 1996;105(5):548-52.

5. Kent G, Minick O, Volini F, Orfei E. Autophagic vacuoles in human red cells. The American journal of pathology. 1966;48(5):831.
