# Supplementary material for: Evaluation of two red cell inclusion staining methods for assessing spleen function among sickle cell disease patients in North-East Nigeria
Source: PLOS Glob Public Health. 2023 May 18;3(5):e0001552. doi: 10.1371/journal.pgph.0001552 (PMC10194925; doi:10.1371/journal.pgph.0001552)
Supplement: Attachment — Submitted filename: RESPONSE to reviewers.docx [file pgph.0001552.s005.docx]

| **Point** | **Query** | **Response** |
| --- | --- | --- |
|  | **Reviewer #1: Comments to the authors** |  |
| Suggestions | - Maybe a short discussion of the findings with regard to a threshold value for the two staining methods, also seen in the light of previous studies, for when to initiate treatment, would be of general interest to the readers | - With regards to specifying a threshold value for both methods, without an existing cut-off value it is difficult to suggest values that be applied in clinical practice for the moment. - However, establishing a baseline for an individual patient and using it to monitor any change in trend may be an option to use to guide when to initiate treatment. - This point has been highlighted under ‘clinical implication’ in the discussion section. |
| 1. | Under Methods:   - “This was a prospective cross-sectional study conducted amongst children and adults with SCD in steady state between September 2020 and November 202; all patients…“. The second year is missing. | - The missing digit has been added under methods. |
| 2. | Under Method for AI count using silver stain: “All stained slides were mounted, as this improved the clarity of microscopy and reduced the effect of reflection observed with unmounted slides”.   - It would be nice to know what mounting medium was used or to have a recommendation. Also the coverslip may be of great importance and should be a #1.5 coverslip (thickness 170 µm) to give the best possible image quality. | - A permanent mount - DPX was used to mount all the AI slides.   The cover slip used: measured 22 x 40 mm with a of thickness 0.13 – 0.16 mm.   - The manuscript has been updated under method for AI count |
| 3. | Under Method for quantitative HJB count using May-Gruenwald Giemsa (MGG) stain:   - Were these slides not mounted? And if they were, with what and with what thickness coverslip etc.? | - No, the HJB were not mounted; mounting did not make a difference to slide clarity. - The manuscript has been updated with this information under method for HJB count |
| 4. | Under Method for AI count using silver stain:” The slides were stained using silver stain at 38C for 20 minutes”.  – The character used in 38⁰C is a 0 not a degree sign. Please revise. | - The manuscript and S.1 appendix have been updated with the appropriate symbol |
| 5. | Under Microscopy: “Microscopy was performed using a light microscope (Leica DM 750, equipped with a ICC50 E colour camera) at 400x and 1,000x magnification”.   - The magnification information should be left out as this does not make much sense (as the magnification varies with reproduction size). Therefore, scalebars in all images, and information on the objectives used is important. - And again, be sure to infer Numerical Aperture (NA) as this tells something about resolution of the objective. | - The manuscript under microscopy   has been updated with the objectives used.   - Although we appreciate the importance of adding scalebars onto the images, however, the microscope was not calibrated at the outset to enable us add scalebars onto the images. - Yes, the numerical aperture for the objectives have been specified in the manuscript under microscopy. |
| 6. | Just a comment for the next study –   - Under Microscopy: A nice test would have been to count the exact same pictures twice, to see the variation between the counts, as this would truly show the intraobserver variation. Two counts of different fields is bound to give different results even at 500 RBCs. | - Thanks. This suggestion is well noted for future studies. |
| 7. | Under General characteristics:   - ” The median ages for the SCD group and controls were 11 years (range 1 - 45 years) and 12.0 years (range 1 - 32 years) respectively.”. Why is the digit left out of the SCD group (11), when it is stated the control age group is 12.0? | - The missing digit has been added to median age of the SCD group and now reads 11.0 years. |
| 8. | Under Identification of argyrophilic inclusion-positive (AI) and HJB red cells :   - “The AI inclusions were spherical, oval or irregular in shape, of variable sizes and occurred either as single or multiple inclusions within a red cell (Fig. 1)”. Could this also say: ”Anything appearing as dark spots or areas in the cells was considered to be AIs”? It just seems to be very random shapes and sizes | - No, only red cells with distinct black granules were counted as positive. The description under microscopy has been updated to make it clearer. - The figure showing AI (Fig.1a) has also been replaced with a clearer one. |
| 9. | Under Comparison of AI and HJB red cells count among the study population:   - It says several times that P=0.000. This cannot be, but most likely is just stated like this, since the value is lower than 0.001. Therefore, it should either say the real value (eg. p=0.000023) or be shown as p<0.001 or p<0.0001. | - The result section of the abstract and main manuscript has been updated with this suggestion. |
| 10. | In Discussion:   - “”…based on that first described by Tham et al in the United States (12).“. Maybe a little awkward to state that it happened in the United States…and remember to put . after al. | - The phrase has been removed from the discussion section of the manuscript and a full stop mark applied after all ‘et al’ in the manuscript |
| 11. | In Discussion:   - “In one study, the authors noted inclusions in 54.3% of red cells from 20 splenectomised individuals, using interference phase-contrast microscopy (21).”. This technique is not phase contrast, but Differential Interference Contrast (abbreviated DIC) and very different from phase contrast. | - This observation has been noted and the manuscript updated with the correct terminology both in the introduction and discussion sections |
| 12. | Under Discussion:  “Kent al found increased red cells with inclusions…”, should be “Kent et al.” | - The citation has been updated under discussion |
| 13. | Under Discussion:   - ”Whatever their nature, it appears the AI are found in increased numbers in SCD patients compared to HJB; the forma although more…”, should be “Whatever their nature, it appears the AI are found in increased numbers in SCD patients compared to HJB; the former, although more…” | - The manuscript has been updated with this observation |
| 14. | Under Discussion:   - “Our data demonstrated a good correlation between the two sets of reading obtained with the AI test (R = 0.90; R2 = 0.82), in keeping with an earlier report (r=0.736; P<0.001)(12).” It is not clear to me, whether this is an intra- or interobserver variation you are considering here – I guess that it is intra? The earlier report mentioned is clearly stated as intraobserver values. Please clarify. - And I do not see any examination of the interobserver variability in the Results section – has this been done? Maybe I just missed it. | - The comparison with the other study was for the intra-observer variation. The sentence has been updated under the discussion section of the manuscript - No, inter-observer variability was not assessed in the current study. Being a new method, and to ensure consistency of the results, only one person read the slides. This point was highlighted under the microscopy section |
|  | Minor comments |  |
| 15. | Under Conclusions:   - “in larger studies may be required for the AI method before the generalizability of its efficacy in assessing spleen function in SCD can be ascertained.”. The z in generalizability indicates US spelling whereas other places, like under Microscopy “(Leica DM 750, equipped with a ICC50 E colour camera)”, colour is spelled as in the UK. Also in Discussion: “In one study, the authors noted inclusions in 54.3% of red cells from 20 splenectomised individuals” – again UK spelling. Please revise and unify. | - The manuscript has been revised to ensure UK spellings are used consistently |
|  | Figures |  |
| 16 | Fig. 1:  The image should have a scalebar and information on the objective used (eg. 40x/0.65 or similar). Not clear if the arrows point to AIs or just cells. Consider white arrows that would stand out from the background better. | - The figure have been updated with information on the objectives used and white arrows used to indicate inclusions - See response to comment number 5 for scale bars |
| 17 | Fig. 2:  Same comments as in Fig. 1 (scalebar and info needed), but here it is clearer that the arrows point to inclusions. Be sure to use same color and size of arrows in both figures. | - The same colour and size of arrows have been used for both figures |
| 18 | Fig 3 and 4.  Since many of the observations lie in the low percentage the graphs are very busy in those regions (especially for the HJB). Consider splitting up both in two showing observations in the 0-10 or 20% range and 10 or 20% to 100% on their own to better show the variation between observers. | - Thanks for this suggestion. We tried plotting the graphs by splitting the values into group of 1-20 and above 20 for the AI data, and into group of <1 and > 1 for the HJB data, however, this did not help improve the clustering of data at the lower end. - We therefore tried improving the plots by log-transforming the data and re-plotting the graphs and there was some improvement, especially with the HJB. - Both plots using the original scale and log transformed data have been included in the manuscript |
| 19 | S Fig 1:  The image is in relatively bad quality, scalebar is missing, information about the microscope objective is needed (both magnification and NA) and it is unclear if the arrows point to AIs or just cells. Black arrows would be better visualized. Please revise accordingly. | - The poor quality of the smear is because of prolonged staining; the reason why it was included in the paper so as to highlight this point. - Information about the objective used has been included and white arrows were used because they provided better contrast with the background |
| 20 | S Figures 2-4:   - Consider putting them together in one figure, making it easier to compare the three. | - The figures have been merged into S.Figs 2A to C. |
| 21 | S1 Appendix 1:   - Under Limitations: The text says “Kent et al found an increase in the percentage of red cells with autophagic”, but the Kent reference is not found in the list of papers, and it should say a year, eg. Kent et al., 1998 (or similar). I later could find the paper in the general reference list. Please put it in the Limitations reference list too. | - The in-text citation and reference list has been updated |
|  | **Reviewer #2: Comments to the authors** |  |
| 22 | - Another control group would strengthen the manuscript (e.g., splenectomized subjects, another hematological disorder). | - Thanks for this suggestion. We will consider adding more control groups in future studies |
| 23. | - In African context, do the authors take in account the malaria parasites? Do the authors have the malaria status of all the participants, as some can be asymptomatic and/or auto-medicated (pyknotic parasites)? | - Yes, the malaria status was obtained from all the participants; this is being reported in another paper focusing on the clinical implication of the current study findings and malaria among SCD patients. |
| 23 | - A clinical dimension is needed. A correlation between AI proportion and spleen status (size and not just presence/absence on ultrasonography) and/or patients’ other clinical data is important to validate the new method and assess the degree of spleen dysfuntion in this population. | - Thanks for this suggestion. It added an important dimension to our study. - We have expanded the analysis between the spleen function markers and various categories of spleen size. - We have updated the methodology, result, and discussion section to reflect this added information |
| 24 | - A little bit more details on AI compositions (nucleic acid? Proteins?) are needed in the introduction even though discussed later (discussion). | - The introduction and discussion have been updated with additional information about the nature of the AI |
| 25. | - In Tham et al., hematopathology, 1996: they reported a greater sensitivity of argyrophilic RBC count over HJB as a test for splenic function, why not look for another marker of spleen function (pocked even though not accessible, it is not impossible for such an impacting study). It would be great to see the correlation with pitted RBC specifically, at least for some participant. | - I agree it would be great to compare the AI test with a more sensitive test like the pocked RBC test, but the lack of availability of this technique and others such as flow cytometry in most low-resource laboratories in Africa is the reason why there is absence of data on splenic function among SCD in the continent. - This was highlighted in the introduction and discussion section of the manuscript. - The primary aim of our current study was to try and demonstrate a test that will be readily available and require less training and equipment, and hence the HJB test was used as alternative for that purpose |
| 26. | - What the authors can propose to improve the working solution stability, as it deteriorates after about 3 minutes of preparation? | - Unfortunately, silver nitrate when combined with the colloidal developer is inherently unstable. We did try to improve stability by using two different brands of gelatin, but this did not result in improved stability of the stain - Although - as outlined in the SOP - , being organised and working quickly meant it was possible to stain up to 40 slides per batch. It was this instability and the better and easier HJB method that led us to recommend the HJB method in preference to the AI one. |
| 27. | - Do the authors think that 500 RBC per smear is enough to reasonably assess the proportion of AI and 400 RBC per smear for HJB? Depending on the field, this count can drastically change. | - Your suggestion is well noted. The choice for the number of RBCs counted was based on the original work for the AI count by Tham et al.      - For the HJB count, there is no specific value used in the literature. We chose to count 400 RBCs in close range to the count used for the AI red cells. |
| 28. | - The authors elaborate about intra-observer variation, what about inter-observer variation? | - **See response to comment number 14** |
| 29 | - If argyrophilic RBC proportions are correlated to pocked RBC proportions (as reported in the discussion), what that tells about the contain of vacuoles in pocked RBC specifically knowing that some vacuoles are empty? | - The possible nature and contents of the vacuoles have been expanded upon in the discussion |
|  | **Minor points** |  |
| 30 | - The first sentence of the methods “study participant”: I guess the enrolment is between “September 2020 and November 2022?” | - **See response to comment number 1**. |
| 32 | In methods “Blood smears”: what explains the discrepancy between the fixing solution in the manuscript and that of Tham et al.,1996   - “Fix a thoroughly air-dried blood smear or a Wright-stained smear for 3 minutes in a solution made by diluting 150 mL of formalin (37% formaldehyde) to 500 mL with 95% ethanol”? | - The choice of 150 to 450 was used in the final protocol to allow for ease of calculating the volume in a 1 to 3 ratio. - This combination was tested during the pilot phase and was found not to affect the quality of the red cells or detection of AI . |
| 33 | - On figures 5, 6a and 6b, horizontal lines (dashed or not) delineating variations must be replaced accordingly | - The figures have been updated; accordingly, bars delineating variations have been inserted |
|  | **Reviewer 3** |  |
| 34 | - A table describing the cohort will be very useful indicating the median and range of age; the sex ratio; the genotype ratio and other hematological parameters including hemoglobin, fetal, hemoglobin, absolute reticulocyte count | - Information on the age ratio is included in the result narration and table one now contains information about the genotype ratio and sex ratio. - Haematological parameters were not included in the current report. These are the subject of a separate paper examining clinical and laboratory parameters associated with splenic dysfunction |
| 35 | - Page 7 line 7, the authors indicate that the setting up of the AI staining was performed using samples of control and SCD patients that were not participants in the study. Where these individuals consented under the ethical agreements indicated in this study? If yes, then the authors should remove the sentence “not participants in our study” as this is misleading? If the individuals were not consented, I do believe we they can’t be indicated in the study. | - Yes, these individuals were consented. - The sentence ‘not participants in our study’ has also been removed from the manuscript for clarity. |
| 36. | - Figure legend of Figure 1 and 2, authors are advised to indicate the genotype of the patient, is he/she SS SC or SB Thal? | - The legends for both figures have been updated accordingly with the genotype information |
| 37 | - Is Table one part of the supplementary, I suggest for it to be part of the main text. - Page 11, it will be very informative to have a histogram representing the %HJB-red cells in the SCD group vs Control group as this will be visually very informative for the readers. - The P value indicated for this comparison is P=0.000, I think a number is missing in the end? Could the authors please verify this? - What statistical test did the authors use? Could the authors also indicate the test used in the text please. - Where all the patients used in this analysis, the number of patients could also be indicated in the text or the figure that I have suggested above. | - No, Table 1 is part of the main text and not supplementary. - We tried representing the information using a histogram, but opted for dot plots as these made the results clearer. - See response to comment number 9 - Non-parametric test was used; this is indicated in the figure legend and statistics in the method section. - The number of participants used in the analysis are indicated in the legends for both plots |
| 38 | - Same comment as above, for the AI red cells, both graphs could be done in one figure. - Same comments as above for the paragraph about the comparison of the frequency of AI and HJB red cells based on the presence or absence of spleen on ultrasonography. The authors could provide a figure with 4 graphs to sum up all the above comments. | - Graphs for comparison of AI and HJB between the SCD and control have been combined as one figure. - Plots for comparison of spleen size and both markers across the study population have been added |
| 39. | - The authors are advised to indicate the statistical test used in the text when appropriate. | - The manuscript has been updated and statistical test used provided with the legends for figures or within the result narrative as appropriate. |
| 40. | - Page 12. The paragraph entitled “comparison of frequency of red cell inclusions using the silver stain and MGG stain method” could be merged with the first paragraph of the results. - The authors could also compare the HJB results to previously published work looking ath HJB red cells in SCD patients (SS patients, El Hoss et al 2019 Blood advances, SC patients: Pourdieu et al 2023, American Journal of Hematology). Even though these papers use flow cytometry-based technique it is rather important to see that the range obtained in this study and with flow cytometry is very similar and this will further provide evidence that the technique used in this study is informative of the HJB-red cell number and thus of spleen function. | - The suggested paragraph has been merged with first paragraph of the results. - Thanks, the suggested references have been highlighted in the discussion section |
| 41. | - Page 12 and 13. Figure 3, 4 and 5 could be merged into one figure, the paragraph of “intraobserver reliability between counts…” is very short and require further elaboration or it could be merged with the paragraph about “intra-observer agreement between counts obtained for the AI and HJB method” and the paragraph about the “comparison of frequency of red cell inclusions using the silver stain and MGG stain” as all of these paragraphs are technical. | - The paragraph of “intraobserver reliability between counts has now been elaborated upon. The four plots showing reliability have been merged. |
| 42. | - Page 17, in the discussion authors are advised to discuss their results to the papers I have suggested earlier, for SS patients, El Hoss et al 2019 Blood advances, SC patients: Pourdieu et al 2023, American Journal of Hematology. Note that the Blood advances paper is a longitudinal study investigating spleen function in SS patients which might be interesting to discuss and compare to the current study of the authors. | - Thanks. The references have been compared to the current work |
| 43. | - Did the authors check if the read out of spleen function correlated with anemia factors that are regularly measured in SCD patients including hemoglobin and absolute reticulocyte count. - Another interesting point will be to study the relationship between spleen function and risk of infection and acute splenic sequestration crisis in this cohort. | - See response to comment number 34 - See response to comment number 23 |
|  | Minor revisions: |  |
| 44. | - Page 4 line 15, the authors could cite reference 8 together with reference 7 for flow cytometry assay as reference 8 has done both imaging flow cytometry and flow cytometry. | - The references have been updated with this suggestion |
| 45. | - Page 6, 2nd line of the “Study participants” the authors write “in steady state between   September 2020 and November 202” the year (in red) should be corrected. | - See response to comment number 1 |
| 46. | - Page 6, blood smears section, the authors indicate the slides were washed with water, I imagine this is deionized water, if yes could the authors amend this in the text. | - The HJB slides were washed with distilled water. The method section has been updated with this information. - The AI slides were washed with tap water as both tap water and deionized were tested during the pre-study phase and there was no difference in slide quality. |
| 47. | - Page 7 line 7, the authors write “normal control”, I highly suggest removing the word “normal” and just keep control. The latter is highly advised to be corrected in all the text | - The manuscript has been updated accordingly |
| 48. | - Page 7 line 21, I kindly ask that the authors indicate the mounting media that was used. | - See response to comment number 2 |
| 49 | - In the methods section, the authors sometimes indicate the company from which the products are purchased from and in other parts the latter is not indicated. I kindly ask that this is homogenized in all the paper. | - This has been noted in the manuscript and the company for all the reagents used have been indicated |
| 50 | - Figure 1 and Figure 2 could be merged into one Figure and labelled Figure 1A and Figure 1B | - The figures have been merged as suggested |
